# Is bureaucracy being busted in research ethics and governance for health services research in the UK? Experiences and perspectives reported by stakeholders through an online survey

**DOI:** 10.1186/s12889-023-16013-y

**Published:** 2023-06-12

**Authors:** Helen Snooks, Ashrafunnesa Khanom, Rokia Ballo, Peter Bower, Katherine Checkland, Jo Ellins, Gary A Ford, Louise Locock, Kieran Walshe

**Affiliations:** 1 Faculty of Medicine, Health and Life Science, Swansea University, Swansea, SA2 8PP UK; 2 Nuffield Trust, 59 New Cavendish St, London, W1G 7LP UK; 3 Division of Population Health, Health Services Research and Primary Care, University of Manchester, Oxford Road, Manchester, M13 9PL UK; 4 Health Services Management Centre, School of Social Policy, University of Birmingham, Edgbaston, Birmingham, B15 2RT UK; 5 Oxford University, Oxford University Hospitals NHS Foundation Trust, Oxford, UK; 6 Health Services Research Unit, University of Aberdeen, Foresterhill, Aberdeen, AB25 2ZD UK; 7 HSR UK c/o Nuffield Trust, 59 New Cavendish St, London, W1G 7LP UK

**Keywords:** Health services research, Ethics, Governance, Online survey

## Abstract

**Background:**

It has long been noted that the chain from identification of need (research gap) to impact in the real world is both long and tortuous. This study aimed to contribute evidence about research ethics and governance arrangements and processes in the UK with a focus on: what works well; problems; impacts on delivery; and potential improvements.

**Methods:**

Online questionnaire widely distributed 20th May 2021, with request to forward to other interested parties. The survey closed on 18th June 2021. Questionnaire included closed and open questions related to demographics, role, study objectives.

**Results:**

Responses were received from 252 respondents, 68% based in universities 25% in the NHS. Research methods used by respondents included interviews/focus groups (64%); surveys/questionnaires (63%); and experimental/quasi experimental (57%). Respondents reported that participants in the research they conducted most commonly included: patients (91%); NHS staff (64%) and public (50%). Aspects of research ethics and governance reported to work well were: online centralised systems; confidence in rigorous, respected systems; and helpful staff. Problems with workload, frustration and delays were reported, related to overly bureaucratic, unclear, repetitive, inflexible and inconsistent processes. Disproportionality of requirements for low-risk studies was raised across all areas, with systems reported to be risk averse, defensive and taking little account of the risks associated with delaying or deterring research. Some requirements were reported to have unintended effects on inclusion and diversity, particularly impacting Patient and Public Involvement (PPI) and engagement processes. Existing processes and requirements were reported to cause stress and demoralisation, particularly as many researchers are employed on fixed term contracts. High negative impacts on research delivery were reported, in terms of timescales for completing studies, discouraging research particularly for clinicians and students, quality of outputs and costs. Suggested improvements related to system level changes / overall approach and specific refinements to existing processes.

**Conclusions:**

Consultation with those involved in Health Services Research in the UK revealed a picture of overwhelming and increasing bureaucracy, delays, costs and demoralisation related to gaining the approvals necessary to conduct research in the NHS. Suggestions for improvement across all three areas focused on reducing duplication and unnecessary paperwork/form filling and reaching a better balance between risks of harm through research and harms which occur because research to inform practice is delayed or deterred.

## Introduction

Despite wide acceptance of the desirability of basing practice and policy in healthcare on rigorous evidence of safety and cost-effectiveness, it has long been noted that the chain from identification of need (research gap) to impact in the real world is both long and tortuous [1, 2]. Initiatives have tried to address hold ups at each stage so that research funding is well spent to deliver research findings that are relevant, timely and implemented to achieve real improvements in care delivered and health outcomes for patients or across populations [3]. Although rapid evaluation and evidence dissemination centres have been commissioned [4, 5] to try to speed up the production of evidence, researchers remain vulnerable to criticism for producing high quality evidence too slowly for decision makers to use that evidence in policy or practice guidance, planning and decision-making [6–8].

Health Services Research (HSR) is a multidisciplinary field that investigates healthcare service organisation, access, quality and costs in order to improve health and well-being of patients and populations [9]. Health care innovations are of particular interest. Many healthcare policies and practices continue to be implemented widely without evidence of effectiveness [10, 11].

In this paper we focus on the links in the chain of research production and implementation which relate to the permissions required to carry out research in NHS settings in the UK. Health services researchers need to gain permissions in order to set up and undertake research with patients, the public or members of staff based in NHS settings – these permissions cover ethical approval; capability and capacity of sites to participate; data protection compliance; and information governance.

**Fig. 1:**
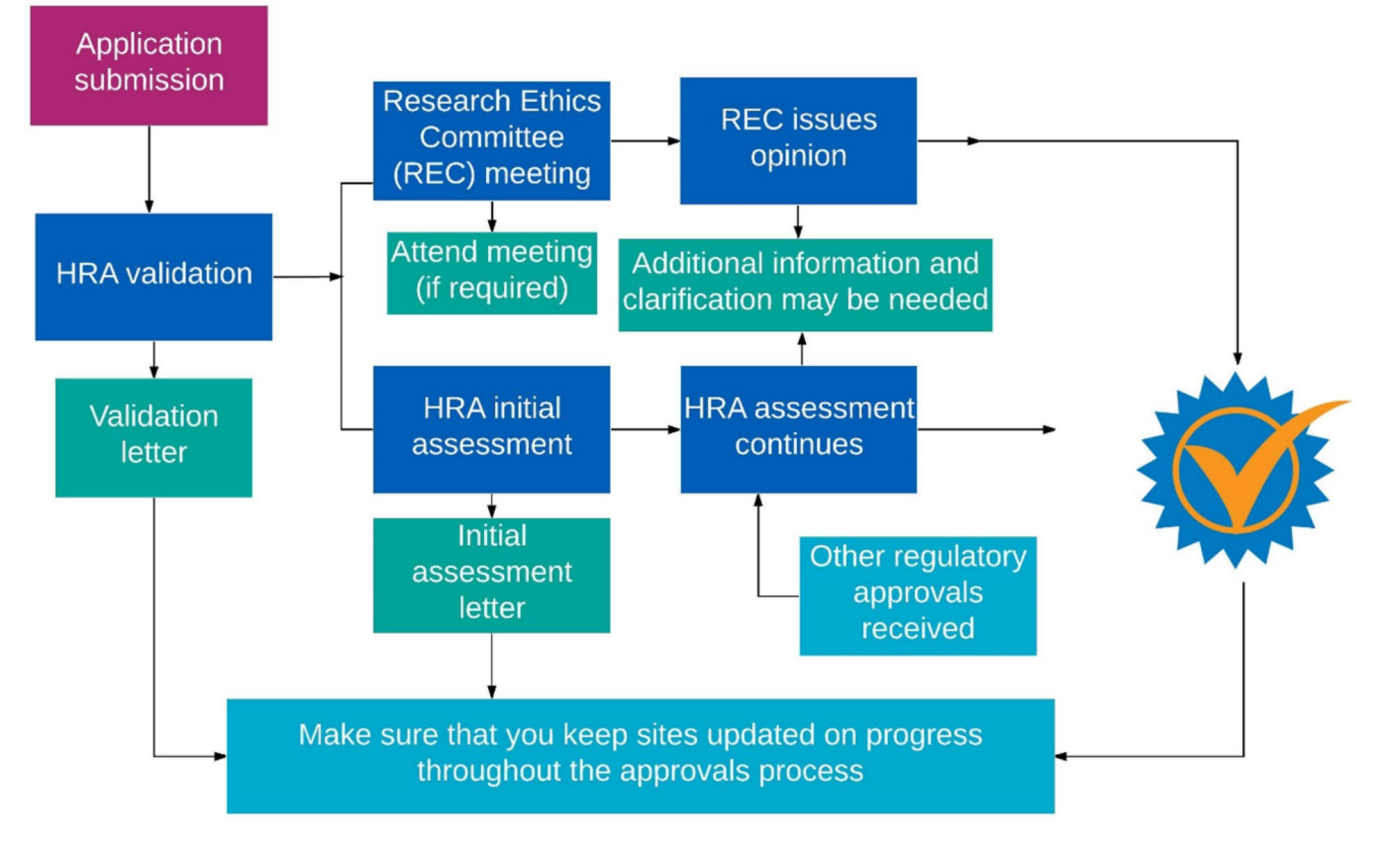
Summary of processes undertaken before starting research [12]

There have been many attempts to streamline processes for ethics and governance in the UK, with the formation of the Health Research Authority (HRA) in 2011. Since 2016 in England, and 2018 in England and Wales, there has been a unified system for applying for approvals for all project-based research in the NHS. Figure 1, below, summarises the processes to be followed before research can start.

## Aims

The aim of this study was to identify and assess the views and experiences of those involved in HSR, within three key areas; research ethics, research governance, and information governance.

On behalf of HSRUK, the authors collaborated with the Health Research Authority (HRA), National Institute for Health Research (NIHR) and other partners to consult with those undertaking or affected by HSR in the UK to understand experiences and views related to:

- What works well
- Problems
- Impact on delivery of research
- Looking forward – improvements

## Method

### Online survey

An online consultation survey was widely distributed from 20th May 2021 and closed on 18th June 2021. Invitations to participants included a request to forward the survey link to others, using a snowball sampling approach. The sample consisted of HSR UK member organisations (n = 41), those on the HSR UK mailing list (n = 4212), and followers of the HSR UK Twitter account (n = 4609), who were actively encouraged to retweet it.

### Questionnaire

The questionnaire was designed by HSRUK Board members and partners (authors HS, KW, RB, AK) to include closed questions related to demographics and role; and open questions related to our study objectives: what works well; problems (if any); impact on delivery (if any); differences during the COVID-19 pandemic; suggestions for improvement in the three domains of research ethics, research governance and information governance.

Closed (categorical) questionnaire responses such as place of work and role were analysed descriptively and are presented without further manipulation. Open (narrative) questionnaire responses were coded thematically within each question and domain. Responses were split by theme so that if one respondent reported several aspects within one response (e.g. delays, stress, costs) this one response would be assigned to three codes. Results are presented by coded comments rather than by respondent so that there may be more coded responses that total respondents in any one question/domain. Quotations are provided to illustrate comments made – and where these varied widely, more quotations are provided to demonstrate the range of responses.

## Results

### Characteristics of respondents

Completed responses were received from 252 people. Over two thirds were based in universities (68%, n = 172) with a further quarter based in the NHS (25%, n = 61). Other respondents reported that they were based in charities (n = 4), the Academic Health Science Network (n = 2), non-NHS healthcare providers (n = 2), local authority (n = 1) and pharmaceutical company (n = 1).

Two thirds described themselves as academic/researcher (66%, n = 164); a further 10% as NHS clinician (n = 24); 7% as research administrator (n = 17); 4% as student (n = 9) and others as clinical lecturer (n = 2), evaluator (n = 2), Patient/Public involvement/engagement person (n = 1), commissioner (n = 1) or Medical Officer (n = 1).

Almost half of respondents reported that they had been named as lead (principal/lead) investigator for externally funded research (41%, n = 148).

Research methods respondents reported they used included (respondents could tick more than one box): interviews/focus groups (64%, n = 160); surveys/questionnaires (63%, n = 159); experimental/quasi experimental (57%, n = 146); analysis of existing routine data (43%, n = 107); observation/ethnography (39%, n = 97); with small numbers reporting involvement in evidence synthesis (n = 2); participatory research/co-production (n = 2); health economics (n = 1); and biomedical research (n = 1).

Most respondents carried out research with patients as participants (91%, n = 229); with 64% reporting that they carried out research with NHS staff (n = 162) and half reporting their research involved members of the public (n = 126). Small numbers reported carrying out research with other (non-NHS) professionals (n = 13); social care users (n = 5); policy makers (n = 4), commissioners (n = 2); carers (n = 1); and other researchers (n = 1).

### What worked well

The availability of national co-ordinated systems was valued across all three areas of ethics and governance (n = 118). Respondents felt that processes were rigorous and well respected (n = 58), giving confidence to researchers that they were following acceptable standards, particularly when research included vulnerable participants (n = 9) (Table 1). Some respondents felt that processes and requirements were clear (n = 50). HRA and other staff were reported to be helpful in supporting development and submission of applications for ethical or governance approval. A minority of respondents reported that systems were well designed and transparent, and applications could be shared between investigators (n = 6).

Some respondents reported that timelines were clear and that guidance (n = 8) is available.

But even in response to this question which sought positive experiences, across the three areas of research ethics, research governance and information governance, there were many negative comments. For instance, the largest category of responses to what worked well in information governance was “nothing” or “very little” (n = 32).

### Problems

This question received the most comments (n = 684 coded comments) (Table 2).

The most frequently reported problem was the complex (n = 139) and bureaucratic nature of the approvals processes with unnecessary duplication of information required (n = 103). Respondents noted that the system is not easy to use, clarity about permissions required is difficult to find – and sometimes seems arbitrary (n = 42). Support was not always available (n = 8), changes to processes were frequent (n = 11) and delays were reported to be extensive (n = 141). Systems and processes were described as inflexible and disjointed.

In particular, many respondents reported that processes are disproportionate to risk for many studies, particularly non-intervention studies, studies using routine data only and qualitative research. Several respondents described the poor fit between a system designed for clinical (randomised) trials and qualitative, participatory or other mixed methods studies which evolve during the conduct of the research (n = 95). The requirement for all study materials to be developed and submitted before any research can begin was reported to be detrimental to collaborative working, particularly with patients or the public, and resulted in the need for frequent amendments to be submitted and approved – a further time consuming process that could again cause delays to study timelines (n = 7).

Respondents reported inconsistent practice between ethics committees (n = 35), resulting in wasted time, frustration and the potential for selection of preferred committees. Related to this was the noted lack of understanding or expertise in clinical, population or methodological areas e.g. palliative care; people with mental health problems; vulnerable groups; observational/routine data/qualitative approaches (n = 17).

Several respondents reported that committees may be out of touch and that some areas of feedback that have become standard practice (e.g. lengthy and complex patient information sheets) exclude participants, particularly people with learning, communication and sensory disabilities as well as other ‘hard to reach’ groups (n = 34).

Respondents reported that there was a lack of clarity around definitions of research (which requires HRA approval) and service evaluation (which can be undertaken without ethical approval) and processes of ethics and governance - what is required, who provides what, where to seek help.

Data sharing agreements were described as very time consuming and challenging to negotiate. Variations in requirements or decisions between partners were reported by 12 respondents.

Several respondents referred to the behaviour of committees and other staff as unreasonable, defensive, risk averse, unfair and even aggressive – leaving researchers distressed and demoralised and impeding progress. Committees were reported to provide comments on study design and other aspects of the research that were felt to be out of their remit (n = 6). Newly introduced financial review processes (e.g. SoECAT – a newly introduced system for allocation of costs) were described as an additional burden (n = 6).

### Impact on delivery

There were over 500 comments provided on impact on delivery – more than half of these comments related to delays (n = 300), sometimes for months or years (Table 3).

Many respondents (n=75) reported that processes for gaining approvals changed or deterred research, innovation and collaboration (n=73), inhibiting the production of research evidence to inform policy and practice (n = 30). For instance, respondents reported that they avoided carrying out studies with patient contact due to the requirement for ethical and governance approvals. Approvals processes were reported to impede or block research as deadlines were missed or researchers ran out of steam.

Many respondents (n = 26) reported high workloads related to the bureaucracy of approval processes, which for some (n = 34) became stressful and affected their morale. The challenge of gaining approvals in order to start and complete research was described in the context of external funding and short-term contracts for many researchers.

### Suggestions for improvement

There were many suggestions for areas to improve, and some suggestions about how this could be achieved (Table 4).

In line with challenges described in previous questions, most suggestions related to streamlining or simplifying HRA and other processes (n = 195), in particular for low-risk research (n = 79). Suggestions about how to do this included the elimination of duplication e.g. between documentation such as study protocol and various sections of the online form; avoidance of complex arrangements; development of triage and potentially different pathways for different types of research; and a flexible approach for study designs which evolve over the period of the research.

Some respondents made suggestions about changing attitudes or overall approach – so that committees are less confrontational, less defensive, and change some assumptions about the behaviour of researchers. Respondents suggested that committees and others involved in ethics and governance processes e.g. Research Ethics Committees (RECs), Confidential Advisory Group (CAG), NHS Digital consider the balance of risks and harms to research participants and those outside the research process who may lose the benefit of the findings, should the research not go ahead or be delayed.

Several respondents asked for more provision of support, clarifications, improvements to online systems and changes to timelines to improve speed of processes.

Respondents suggested ensuring that committee membership represented the populations served, through e.g. Equality Assessment processes. Virtual processes and meetings were appreciated by some, whilst others wanted to see a return to face to face meetings.There was general agreement that inconsistencies need to be addressed e.g. through provision of standardised templates and guidance.

**Table 1:** What works well in health services research

Theme	Total across areas n	Research ethics n	Research governance n	Information governance n	Key quotations
National co-ordinated system in place	118	56	46	16	One system to access information on all approval forms and submit applications … is good (RE)
Robust	58	24	22	12	The external assurance granted that research has been thoroughly assessed and deemed legal and ethical should not be underestimated (IG)
Helpful, friendly, supportive staff	59	14	30	15	Individuals handing the applications tend to be helpful and supportive (RG)
Clarity	50	20	19	11	The quick HRA check about whether ethical approvals are required (RE)
Speed/ timeliness	28	22	6	-	There is a timely response once the application is submitted (RE)
Online/ virtual process	22	22	-	-	Thorough online systems accessible from home, by different project members (RE)
Availability of guidance	15	7	-	8	The guidance and support has been clarified to a much better standard recently (IG)
Proportionate review	15	15	-	-	Having a fast-track system for low-risk research is helpful, except that the bar is far too high (RE)
Feedback strengthening study	14	14	-	-	Feedback from ethics committees can be very helpful in shaping/refining/improving projects (RE)
Good local relationships	12	-	12	-	I know the people I need to work with (RG)
Availability/ choice of RECs	10	10	-	-	Online booking systems; seeing REC meeting dates online (RE)
Gives confidence standards met	9	9	-	-	It acts as a safeguard for vulnerable people (RE)
Consistency	8	8	-	-	It applies a ‘yardstick’ across all studies, ensuring uniformity and consistency which is important (RE)
Low burden on service providers	8	-	8	-	Lower administrative burden for Trusts. HRA approval letter provides clear instruction for Trust (RG)
Sponsor support	7	-	7	-	We have a great research department who are all well versed in research - academic, healthcare, government and commercial (RG)
Improved	6	6	-	-	Speed of panels/committees much quicker than before (RE)
Data availability	6	-	-	6	Data coverage is continually improving (IG)
User friendly system	6	-	-	6	Regional systems have streamlined governance and it is possible to amend applications to ask for additional years of data without going back to the start (IG)
Simple quick process for amendments	5	5	-	-	Simple amendment system to add additional sites etc. (RE)
Responsive	5	-	5	-	HRA are generally quick to respond and approve low risk studies (RG)

Abbreviations: RE - Research Ethics, RG – Research Governance, IG - Information Governance.

**Table 2:** Problems identified by respondents

Theme	Total across areas n	Research ethics n	Research governance n	Information Governance n	Key quotations
Lack of integration of systems	139	49	85	5	Endless stream of middle managers in different organisations requiring the same information from me, not trusting information given elsewhere and not being in a position to make decisions (RG)
Delays/ lengthy process	141	49	66	26	The lead time required to obtain data from NHSD precludes a great deal of responsive, policy-relevant research (IG)
Bureaucratic/ repetitive/ laborious	103	56	13	34	The IRAS form is far too long and is really onerous to complete. So much of the detail requested is available in the protocol and patient information materials so it is just repetition. Why submit your protocol and repeat it all in a form? …. All of this unnecessary admin just delays submission (RE)
Disproportionate for low risk studies/ inflexible	95	71	18	6	So many forms and boxes to complete for ethics for a simple qualitative interview study. The whole system started with RCTs and has never really moved beyond them (RE)
Inconsistent	35	23	-	12	Unwarranted variance in interpretations of differing Information Governance teams (IG)
Out of touch/ unethical/ excluding	34	22	12	-	Procedures often exclude vulnerable people due to definitions of capacity whereas participatory research takes consent as a process throughout the research .… there are no tiered procedures that enable applicability to the research at hand. One size doesn’t and can’t fit all. (RE)
Lack of clarity	42	21	-	21	Poor understanding of the law around IG, resulting in conflicting advice and policies even within the same organisation. (IG)
Attitudes of staff/ committees: risk averse/ defensive/ aggressive	21	5	16	-	The defensive attitude and slowness of many R&D departments, and their ability to make you feel like you’re a dangerous threat (RG)
Lack of specialist understanding/expertise	17	17	-	-	Lack of knowledge from RECs about research on sensitive topics e.g. palliative care (RE)
Data sharing agreements	14	-	-	14	IG departments can be very slow to process applications to conduct research. Data sharing across NHS Trusts can be extremely bureaucratic (IG)
Expensive	13	4	-	9	Expense of accessing datasets sometimes means that research is not feasible (IG)
Frequent changes to system	11	7	-	4	Every time I come to another project the process and the forms have changed yet again so you can’t even used what you learned last time to help you (RE)
Availability of help/support	8	8	-	-	Challenges in finding the right person to speak with about queries or indeed finding anyone (RE)
Additional delays for amendments	7	-	7	-	The fact that you have to get C&C again with every amendment is a complete nightmare. (RG)
Student research requiring more guidance	7	-	7	-	Student research applications not meeting NHS HRA standards. Lot of resource invested in explaining the system and referring students back to their HE to refine their applications (RG)
Not sticking to remit	6	6	-	-	Ethics committees requesting changes to format and designs, which has nothing to do with ethical considerations (RE)
Poorly designed system/not user friendly	6	6	-	-	The IRAS forms are not easy to complete as you can only see two lines at a time! (RE)
Different processes for research/ service evaluation/ improvement	6	6	-	-	The definition of the distinction between [service evaluation or research].does not make sense and is not consistent between University and NHS documentation, leading to the risk of game-playing (RE)
Financial review additional burden	6	-	6	-	SoECAT not being accepted despite hours of work creating and getting approval. Pharmacy delays, additional local documents being requested, individual departments asking for funding despite SoECAT and saying that they don’t see any research funding. (RG)
Pedantic	5	5	-	-	Minor changes being requested to documentation which are not really needed (RE)

Abbreviations: RE - Research Ethics, RG – Research Governance, IG - Information Governance.

**Table 3:** Impacts on research delivery

Theme	Total across areas n	Research ethics n	Research governance n	Information Governance n	Key quotations
Delays	300	121	110	69	There are delays to research even though there is no flexibility on funding (RE)
Deters, restricts or changes research, innovation/ collaboration	73	46	21	6	We have avoided setting up studies, compromised on our sampling strategies and generally been discouraged. The general feeling is that HRA should be avoided if possible (RG)
Workload/ difficulty/ waste	75	26	34	15	Major administrative burden (IG)
Reduced quality	30	18	-	12	Perhaps the most pernicious impact is the fact that every time you want to change a sentence on a leaflet you have to go through an amendment, which is more paperwork and more delays. It basically means that you don’t bother changing things even if it would improve the study/recruitment/participant experience and the research is of lower quality as a result (RE)
Research delivery/ performance	30	17	-	13	My funding ran out while I was waiting for the data to arrive, so now I cannot do anything with it. (IG)
Staff stress/ morale	34	15	14	5	Massive, catastrophic: it now takes up more time than the research itself sapping the will to live let alone motivation to undertake research. (RE)
Increased costs	20	12	-	8	The main impact is to raise the cost of the work, as researcher time is invested in negotiating a byzantine process of form-filling with significant invisible costs which are, inevitably, borne by the research funders (RE)
Planning burden/uncertainty	8	8	-	-	Timelines unknown so can’t progress things and indicate to sites when amendments will be rolled out as unknown when approvals will be received (RE)
System problems	7	7	-	-	Complications when submitting initial application and any subsequent amendments as comments from more than one committee can be baffling (RE)
Inability to inform policy with timely evidence	8	-	-	8	Massive, massive delays …. consequently, investment decisions continue to be made, without any information about effectiveness. (IG)

Abbreviations: RE - Research Ethics, RG – Research Governance, IG - Information Governance.

**Table 4:** Suggestions for improvements

Theme	Total across areas n	Research ethics n	Research governance n	Information Governance n	Key quotations	
Streamline/ simplify/ standardise	195	97	69	29	Reduce information in forms, use documents submitted and protocol for key information rather than repeating in form (RE)	
Make proportionate /better fit for non-RCTs	79	52	27	-	Remove the need for so many R&D approvals for low risk studies, especially at the boundary of research and audit or research and quality improvement (RG)	
Change of attitude/ approach	41	26	-	15	A change in attitude and culture that aims to be more permissive and enabling – focussing on how the use of data for public good as opposed to what you can’t do (IG)	
Support/ guidance	44	18	12	14	More worked examples to help you complete applications (RE)	
Faster turnaround/ time targets	20	15	5	-	Speedier review for urgent studies without detriment to non-urgent research (RE)	
Clarification	42	11	19	12	Clear understandable requirements so you know if you are meeting the regulations or not (IG)	
Learn from experience	15	15	-	-	It would be good if pragmatic approach that has been used during the pandemic is continued after things start to return to normal (RE)	
Expertise/ representation	12	6	-	6	The road to greater inclusivity has many components and the ethics committees can have a more prominent role in this. It is important to take steps that ethics committees are truly representative of the community – with representatives at senior levels from a broad range of minority ethnic groups (RE)	

Abbreviations: RE - Research Ethics, RG – Research Governance, IG - Information Governance.

## Discussion

### Summary of key findings

The need for high quality rapid responsive HSR has never been greater given the impact of the pandemic on the NHS. This survey demonstrates that the HSR community considers there are major problems with current ethics, governance and IG approval processes applied to HSR across the NHS, and many opportunities for the system to be more streamlined, flexible and responsive.

Aspects which were reported to work well were: centralised systems e.g. IRAS; confidence in having the approval of a rigorous and respected system; helpful staff. Workload, frustration and delays related to processes which were viewed as overly bureaucratic, unclear, repetitive, inflexible and inconsistent were reported as the main problems across research ethics, governance and information governance. A theme raised across areas was the disproportionality of processes for relatively low-risk studies such as some non-interventional, qualitative, and routine (existing) data studies. Assessment of risk needs to be undertaken regardless of methodology, but current processes seem to default to an onerous and one-sided consideration of risk even for low-risk studies. Some requirements were reported to have unintended effects on inclusion and diversity, and to be a very difficult fit with Patient and Public Involvement and engagement processes. Inflexibility, the need to have everything ready at the outset with every small change requiring a lengthy amendments process and overlong, complicated Patient Information Sheets were highlighted again and again as off-putting, particularly for participants in marginalised groups. Existing processes and requirements were reported to cause stress and demoralisation for those involved in trying to produce research, particularly as most research is contracted for fixed time periods, and many researchers are employed on fixed term contracts. Impact on research delivery was reported to be high, in terms of timescales for completing studies, deterrence of research, particularly for clinicians and students, quality of outputs and costs. Many suggestions were made for improvements in each section of the questionnaire, related to system level changes / overall approach and specific refinements to existing systems. Many suggestions were made about how to try to streamline and integrate systems in order to reduce workload and speed up processes for approvals. Key players responsible for these systems - and therefore for change - include the HRA and its component parts (RECs, local R&D Offices, regional Clinical Research Networks); CAG and NHS Digital.

### Study limitations

In this online survey we used a snowball approach to try to gain views and experiences from a wide range of people working in HSR in the UK. Because of this approach we do not have any data about response rate or representativeness of respondents. We used mainly open-ended questions which resulted in a large amount of narrative data to code. We discussed and validated codes to provide a descriptive analysis to present results in a coherent manner, and we carried out one level of coding only.

This survey captured mainly the views of researchers – end-users - rather than those involved directly in the various regulatory agencies.

### Implications of findings

In order to thrive in the long term, research needs to be carried out responsibly, sustainably and efficiently. Structures and processes to gain permissions to undertake research in NHS settings have been developing over the last thirty years. Despite many efforts to streamline these structures and processes, there have been concerns that the regulatory journey for HSR studies has resulted in over complex, duplicative pathways that can cause delay to initiation and completion of studies and are costly to follow [13]. We have found that despite repeated attempts to streamline and integrate permissions processes for research in the NHS in the UK, researchers report that processes and systems remain bureaucratic, with long delays and high financial and personal costs. During the COVID-19 pandemic the need for research to underpin healthcare provision was even more urgent than usual due to the unprecedented volume of demand by patients who were extremely sick and lack of previous evidence or experience of this virus – risk factors; epidemiology; effective treatments; means of prevention (vaccinations) and optimal health care organisation. Changes were made to health research permissions processes in order to expedite COVID-19 related research [14, 15], with mixed success. The NHS setting offers unrivalled opportunities for health research in the UK, but complex structures and processes threaten the ability of researchers to provide timely evidence to inform policy and practice, with systems designed for high-risk interventional research not fit for purpose for low-risk studies. Findings from this survey indicate that current processes for ethical and governance approvals are not only inefficient but have impacts on speed, inclusion and capacity which fly in the face of key policy and funder objectives. Current efforts to ‘bust bureaucracy’, [16] including the current HRA initiative “Think ethics” [17] must: include a new approach to risk assessment across the whole system; reduce complexity; and improve integration of different parts of the overall system for ethics and governance in health services research. Only then can we make strides towards timely production of evidence to inform policy and practice in the UK and internationally.

## Data Availability

The datasets used and/or analysed during the current study available from the corresponding author on reasonable request.
